# Work experience of breastfeeding nurses returning to work after maternity leave in Liaoning Province of China: A qualitative study

**DOI:** 10.1002/nop2.2157

**Published:** 2024-04-17

**Authors:** Xin‐li Wan, Jia‐yi Yang, Ying‐li Pan

**Affiliations:** ^1^ Cardiothoracic Surgery Ward Yichang Central People’s Hospital Yichang China; ^2^ First Affiliated Hospital of China Medical University Shenyang China; ^3^ Fourth Affiliated Hospital of China Medical University Shenyang China

**Keywords:** breastfeeding, management, nurses, return to work, working women

## Abstract

**Aim:**

With the implementation of China's three‐child policy in 2021, the nurse population faces an increase in the number of breastfeeding nurses returning to work after maternity leave. This study aims to describe the work experience of breastfeeding nurses returning to work after maternity leave.

**Design:**

A qualitative descriptive design.

**Methods:**

The data were collected through semi‐structured interviews with eight nurses and analysed through Braun and Clarke's thematic analysis.

**Results:**

Three themes and nine sub‐themes emerged from the analysis of the interviews: changes in nurses (emotional changes, physical changes and changes in work); needs for an improving work environment (needs for a supportive workplace and nurse shortage); support for breastfeeding nurses (support from coworkers, support from the manager, support from the organisation and own need for work).

**Public Contribution:**

This study highlighted that breastfeeding nurses need an adjustment period when they return to work after maternity leave. Successful breastfeeding requires support from coworkers, managers and the organisation. In addition, workplace support for breastfeeding and management for nurses needs to be improved.

## 1 INTRODUCTION

Seventy per cent of global health and social workers are women and nursing and midwifery occupations account for a large proportion of them (WHO, 2022). By the end of 2020, China had 5.02 million registered nurses (National Bereau of Statistics, 2022), with nurses of childbearing age (18–45) accounting for 85.5% (National Bereau of Statistics, 2021). After China implements the three‐child policy in 2021, the nursing population, mainly of childbearing age, will see an overlap in childbirth and the number of nurses returning to work will gradually increase.

The large population of breastfeeding nurses after maternity leave deserves even more attention. China has introduced relevant policies to protect breastfeeding professional women, for example, they are given 1 h to breastfeed during working hours and cannot be assigned to night shifts until the child is 1 year old (The Central People's Government of the People's Republic of China, 2012).

## 2 BACKGROUND

Working women including nurses returning to work after maternity leave are a group of great concern in all countries and all fields. The increased role of mothers after childbirth leads to work‐family and other conflicts (Wiens et al., 2023) and working women strive to integrate multiple identities (Maviso et al., 2022). As they return to work after a long period of absence, they need to be ‘resocialised’ (Sousa & Gonçalves, 2019). Women need to adapt to new changes in work and life, including re‐familiarising themselves with work content, caring for children (Garcia & Viecili, 2018) and adjusting their sleeping time habits (Kawashima et al., 2022).

Working women are breastfeeding when they return to work after childbirth. Although breastfeeding is recognised as the most beneficial feeding method for infant growth and development worldwide (WHO, 2003), the breastfeeding rate in many countries still needs to be improved (Neves et al., 2021). In 2019, The global breastfeeding rates were 88.7% for 6‐month‐old children and 81.1% for 1‐year‐old children (Neves et al., 2021). However, it was worth noting that the overall prevalence of breastfeeding for working women after returning to work was 25% (Dutheil et al., 2021). Therefore, to increase breastfeeding rates, researchers have conducted qualitative and quantitative studies to dig deeper into the methods. After analysing the breastfeeding experiences of working women returning to work, it was found that mothers had negative experiences, lack of privacy protection when pumping, an embarrassment of overflowing milk, conflict with managers over breastfeeding time and hostility of employers towards breastfeeding (Litwan et al., 2021). Working women need breastfeeding support in the workplace, such as the need for breastfeeding rooms with privacy, refrigerators and flexible breastfeeding times (Jiravisitkul et al., 2022; Mao et al., 2018), as well as daycare services (Ma et al., 2021). Alternatively, support for breaks by coworkers and managers is an important part of working women's breastfeeding (Ross & Woszidlo, 2021).

Since there are differences in work culture and breastfeeding between different countries (Dutheil et al., 2021), our study aims to describe the work experience of breastfeeding nurses in a Chinese cultural context returning to work after maternity leave. We believe that such research is important to fully understand the difficulties of breastfeeding nurses and to lay the foundation for further development of nursing management programs.

## 3 METHOD

### 3.1 Design

This study used a qualitative descriptive to describe the work experience of breastfeeding nurses returning to work after maternity leave. Qualitative description is based on naturalistic inquiry and can help us directly describe the experience of the participants (Sandelowski, 2000).

### 3.2 Study setting

The study location is Shenyang, the capital city of Liaoning Province, located in the northeast of China. The specific study site is a third‐class A hospital, covering an area of nearly 120,000 square metres, with 1800 beds and 925 nurses.

### 3.3 Participants

The purposive sampling method was used to select participants. Participants were eight Chinese breastfeeding nurses who returned from maternity leave within 6 months, differentiated by age, education, professional title, years of working, department and gestation. All participants worked at the same tertiary hospital in Liaoning Province of China. The sample size was determined according to the data saturation principle. Data collection was done in parallel with data analysis until no new concepts emerged and data collection can be stopped. Demographics and related information are present in Table 1.

**TABLE 1:** Demographic descriptive of participants (n = 8).

Code	Age	Nation	Education	Professional title	Years of working	Department
Nurse 1	34	Han	Bachelor's degree	Primary nurse	10	Gastroenterology department
Nurse 2	31	Mongolian	College degree	Primary nurse	9	Haematology department
Nurse 3	32	Manchu	Bachelor's degree	Primary nurse	9	Endocrinology department
Nurse 4	32	Han	Bachelor's degree	Nurse	10	Gastrointestinal surgery department
Nurse 5	31	Han	Bachelor's degree	primary nurse	9	Breast surgery department
Nurse 6	30	Han	College degree	Primary nurse	7	Paediatric outpatient department
Nurse 7	27	Han	Bachelor's degree	Primary nurse	7	Emergency department
Nurse 8	34	Han	College degree	Primary nurse	11	Emergency department

### 3.4 Data collection

All data were collected through face‐to‐face interviews with participants from November 2021 to March 2022. One researcher conducted one‐on‐one interviews with the participants individually, in the nurses' lounge and the interviews were conducted during non‐working hours. The interview location was comfortable and quiet enough for the participant to communicate at ease and the interview time was sufficient and did not interfere with the participant's work. The semi‐structured interviews lasted between 30 and 60 min per participant.

The researcher was a female and a student pursuing a master's degree in nursing. At the time of the study, the researcher was in the status of an intern and there was no close relationship or any conflict of interest with the participants. The researcher was trained in a course on qualitative research methodology.

Interview techniques such as rhetorical questions, repetition and follow‐up questions were incorporated as appropriate in the interview process to obtain more comprehensive information. The interview questions were identified after three times pilot tests which are listed in Table 2. Field notes were completed after interviews including the date of data collection, study area, the number of participants, significant nonverbal behaviours and the researcher's critical reflections.

**TABLE 2:** The questions of the interview.

The questions of the interviews
How was your work experience when you returned to work after maternity leave?
What difficulties did you encounter during work?
How did you resolve them?
Are there any other comments you haven't mentioned about your work experience?

### 3.5 Data analysis

The researcher transcribed the audio recordings of the interviews verbatim into text for import into Nvivo12.0 software within 24 h of each interview. The data were analysed using Braun and Clarke's thematic analysis (Braun & Clarke, 2006). Step 1: The researcher read the data repeatedly and recorded initial ideas. Step 2: All data were systematically coded and relevant data were organised to form initial codes. Step 3: Themes were identified. Step 4: Themes were checked for relevance to the whole data. Step 5: Clear definitions and names were generated for each theme. Step 6: An analysis report was produced. In the Nvivo 12.0 software, manual coding was used to create nodes by free coding the textual information related to the study content in each interview text. The same type of nodes were then used as child nodes to create new nodes, thereby forming themes and sub‐themes.

### 3.6 Ethical considerations

This study was approved by The Fourth Affiliated Hospital of China Medical University Ethics Committee (Decision Number: EC‐2022‐KS‐002). Under the premise of following the principle of confidentiality, numbers were used for all participants, the interviews were recorded and the emotional changes of the participants were carefully observed and recorded. The same researcher completed all interviews.

## 4 RESULTS

The eight participants were aged 27–34, three of whom were from ethnic minorities and five from Han Chinese and their education included bachelor's degrees and college degrees. The working years of the participants ranged from 7 to 11 years. Their working departments included medical departments, surgical departments, emergency rooms and an outpatient department. Nurses described work experience and needs during returning to work after maternity leave. Based on this, 3 themes and 9 sub‐themes were identified (Table 3).

**TABLE 3:** Overview of the topics that emerged from the analysis.

Themes	Sub‐themes
Theme 1: Changes in nurses	Emotional changes Physical changes Changes in work
Theme 2: Needs for an improving work environment	Needs for a supportive workplace Nurse shortage
Theme 3: Support for breastfeeding nurses	Support from coworkers Support from the manager Support from the organisation Own need for work

### 4.1 Theme 1: Changes in nurses

The participants' own emotions and physiology changed because they were breastfeeding. Moreover, after maternity leave, the participants found another change was from work.

#### 4.1.1 Sub‐theme 1: Emotional changes

Now I always remind myself that I can't make a mistake when I'm sticking needles. I'm just more careful because, after all, I haven't worked for a long time. (Nurse 6)

Because I was taking care of my baby all day before I returned to work, now that I'm suddenly away from her, I'm worried that the child will not adapt to my absence. (Nurse 3)

Participants displayed anxiety about their work and concerns that they would not be able to do their jobs as well as before. Before going back to work, the participants were typically the babies' major caretakers. However the caregivers changed as a result of their need to go to work, the participants showed worries about the babies' eating, sleeping, physical health and ability to adjust to their absence.

#### 4.1.2 Sub‐theme 2: Physical changes

Because I have to get up three or four times at night to feed my child and work during the day, I'm really tired. (Nurse 8)

My kid wakes up and wants to play with me during the night, which delays my sleep for an hour or two and I don't sleep well. (Nurse 2)

My inability to remember things was particularly evident in the early days of my return. (Nurse 8)

I don't know if it's because the pelvis didn't recover after giving birth, or because of the anesthesia during childbirth, but my back gets a little uncomfortable from time to time. (Nurse 3)

The participants' quality of sleep was significantly impacted by the frequent night‐time interruptions due to the necessity of feeding their infants. Participants felt fatigued while working because of worse quality sleep at night, creating a vicious cycle. Participants admitted that they were still experiencing the aftereffects of childbirth, occasionally experiencing hand or back pain while working. Additionally, participants frequently reported having memory loss and believed this might be because they had to focus so much of their attention on raising children.

#### 4.1.3 Sub‐theme 3: Changes in work

It may be because of the long rest at home, I work slower than I used to be. (Nurse 7)

Now I have to take time to express breast milk twice a day and when I go back to work, I need to go over the work again. (Nurse 8)

We have a lot of exams, and I can only study after the baby is asleep, or I will read in the middle of the night while expressing breast milk. (Nurse 1)

Participants generally reported that due to the long break, there were uncomfortable conditions upon returning to work, including being inadaptable to the work routine and feeling rusty and unfamiliar with the new instruments, knowledge, medications, system and layout. In contrast to previous jobs, lactation also requires expressing breast milk during work, which can interrupt the continuity of work. After the end of the expression of breast milk, the progress of the work changed somewhat and the participants needed to make a new nursing clinical decision. Because caring for children distracted most of their energy, the original career plan could only be put on hold.

### 4.2 Theme 2: Needs for an improving work environment

Participants described the inconveniences that breastfeeding nurses felt in their work.

#### 4.2.1 Sub‐theme 1: Needs for a supportive workplace

I work in an emergency room and am exposed to patients with complex conditions. Because I am so worried about germs being brought home with me, I always shower before I touch him. (Nurse 8)

Now that it's winter, I'll just keep the milk outside the window. When summer comes, I'll carry a heavy storage bag every day and that's a disaster. (Nurse 6)

There is no place to offer me, I could only express breast milk in the lounge, where my coworkers usually have lunch. (Nurse 2)

Many foods in the hospital canteen are not suitable for me. If I eat something wrong, my son will have blood in his bowel movement. (Nurse 5)

All participants expressed breast milk in the workplace and then took it home to feed to their children after refrigerated storage. Most nurses worried about occupational exposure, thinking they were spreading germs, viruses and radiation to their children whose immune systems were still developing, especially with the widespread presence of COVID‐19 in the world. Nurses placed a high value on every part of the food of the kids in the hopes that they will grow up healthy. On the one hand, participants who were contraindicated voiced unhappiness with the food in the hospital canteen, which did not consider their demands. On the other hand, the participants gave a lot of weight to the facilities for storing breast milk and the cleanliness of the expressed breast milk space.

#### 4.2.2 Sub‐theme 2: Nurse shortage

When the work is particularly busy, I must delay expressing breast milk, so I have to endure the uncomfortable feeling. I always worry about work while expressing breast milk, so I just want to do it quickly. (Nurse 5)

At the beginning, the manager would try to arrange for me to do easier work. But then she can't do that because the nurses with lower seniority could not do the job as well as me. (Nurse 2)

Some participants mentioned conflicts between breastfeeding and work. First, there were scheduling issues between work and breastfeeding time. Sometimes, the nurses had to put up with the unpleasant milk increase and the worry of mastitis because they were too busy to express breast milk. Participants were impatient to finish expressing breast milk even during breaks from work because they were concerned about changes to their jobs while they were doing it. Second, there was a conflict between nurses who had not returned to optimal work status and a high nursing workload. Most breastfeeding nurses were the mainstay of the unit and even after maternity leave, their work capacity was still more enhanced than that of nurses of lower seniority. The manager wanted the experienced breastfeeding nurses to undertake the main work as soon as possible, but they had insufficient energy to meet the work demands.

### 4.3 Theme 3: Support for breastfeeding nurses

Participants were supported in many ways upon returning to work after a long leave of absence.

#### 4.3.1 Sub‐theme 1: Support from coworkers

My coworkers will change positions for me to give me time to express breast milk. (Nurse 7)

Whenever I have a problem taking care of my children, my coworkers will discuss together and pass on their experience. (Nurse 8)

The support of co‐workers is not only performed at work but also in taking care of the children and keeping the participants physically and mentally healthy. Co‐workers helped participants with their work when they needed to express breast milk, which relieved the stress of the participants' work and expressing breast milk. New mothers often showed helplessness in caring for their children and co‐workers gave support of knowledge and experience.

#### 4.3.2 Sub‐theme 2: Support from the manager

We can choose the rest of breastfeeding leave according to our circumstances. Anyway, it's an hour and I'm saving my time for a day. (Nurse 2)

The manager will arrange for us to work in the triage positions for a while so that we could get used to the environment and our strength could gradually recover. Although I am quite busy now, I was not as tired as others. (Nurse 6)

My manager and coworkers will not be very concerned about my half‐hour of expressing breast milk. (Nurse 1)

The good working atmosphere created by the manager and coworkers was important for the participants. The humanised management style can make the nursing team more cohesive.

#### 4.3.3 Sub‐theme 3: Support from the organisation

I think the greatest care that the nursing organization takes for me is the breastfeeding leave. (Nurse 1)

Participants expressed that the organisation could fully understand them, showing strong tolerance. Participants thought it was good for nursing organisations to implement breastfeeding leave and no night shifts by regulations.

#### 4.3.4 Sub‐theme 4: Own need for work

I don't like children. When I go to work, it is like a vacation. (Nurse 1)

Participants generally felt that taking care of their children at home was more tiring than work and preferred to work, believing that work played a very important role in their emotional regulation.

## 5 DISCUSSION

This study sought to explore the work experience of breastfeeding nurses who returned to work after maternity leave. Inductively, we found that breastfeeding nurses faced some changes and needed an improving work environment and they felt support from coworkers, managers, organisation and self.

Working women returning to work after maternity leave find it difficult to reintegrate into society (Li et al., 2023). Passing the adjustment period of returning to work requires not only nurses themselves to make proactive adjustments but also nursing managers to help them overcome the difficulty of returning to work (Costantini et al., 2022). Cátia Sousa's study showed that difficulties were mainly focused on reducing the time allocated to family, restoring routine and work rhythm and the work environment (Sousa & Gonçalves, 2019), which is consistent with the results of this study. In contrast to the results of Cátia Sousa's study, managers in our study took into account the nurses' situation when scheduling their work, for example, by temporarily placing them in support or other jobs for some time to help them gradually adapt to their work without overexert. Reducing nurses' work stress can help nurses return to work through the adjustment period (Kokubo et al., 2023). In addition, work–family support policies can partially moderate work–family conflict and can reduce the negative impact of work–family conflict on positive work attitudes (Costantini et al., 2020). Therefore, in the future, an in‐depth study can be conducted on how to develop a standardised management method to help breastfeeding nurses through the return‐to‐work adjustment period.

Although breastfeeding leave for working women was guaranteed in policy, our study found that nurses also needed a workplace that supports breastfeeding, such as a concealed expressing breast milk room and equipment for storing breast milk. The participants in our study had no expressing breast milk dilemma, which may be related to the small sample size. In Mexico, few workplaces offer express breast milk rooms, resulting in some working women having to express breast milk in toilets, conference rooms, closets or cars (Hernandez‐Cordero et al., 2022). A survey of working women in the United States found that women with space and time off to express breast milk were more likely to be exclusively breastfeeding at 6 months of age (Kozhimannil et al., 2016).

Workplaces providing expressing breast milk rooms can not only help nurses prolong breastfeeding time to achieve personal breastfeeding goals (Goulden et al., 2022) but also have a positive impact on the new type of breastfeeding performed by nurses (Wallenborn et al., 2019). A study found that women who felt supported in the workplace for breastfeeding had higher overall job satisfaction (Thomas et al., 2022). More importantly, workplace support can help working women achieve work–family balance (Wiens et al., 2023).

The workplace is a space to promote breastfeeding and protect breastfeeding nurses (Vilar‐Compte et al., 2021). Accordingly, providing a breastfeeding‐friendly environment for nurses who are breastfeeding in the workplace is still an issue worth exploring.

Managers' and coworkers' support is important to breastfeeding nurses. Our study found that manager support was demonstrated by the nurses' ability to flexibly schedule their breastfeeding during the workday according to their preferences and coworkers' support was mainly demonstrated by sharing the workload while the nurses were breastfeeding. However, other studies found that there was a lack of support from managers and coworkers to be afraid to breastfeed. This may be because managers are concerned that breastfeeding nurses are less productive and need more time off and that other nurses view breastfeeding breaks as special treatment and cause resentment. However, in the long run, managers' support of breastfeeding has long‐term benefits in terms of helping to recruit and retain nurses (Chang et al., 2021).

Coworkers and managers consider it unprofessional to expect co‐workers to undertake their work when they are expressing breast milk and frequent breastfeeding breaks by women during working hours could lead to feelings of inequity among coworkers (Porter, 2017). Wambach's study showed that nurses felt the most support from their coworkers at work (Wambach & Britt, 2018). Previous studies showed that support from coworkers and managers was beneficial in prolonging breastfeeding and even sometimes support from coworkers was more important than support from managers (Cervera‐Gasch et al., 2020). Coworkers' support for breastfeeding influences their willingness to continue breastfeeding (Jantzer et al., 2018). Zhuang's study showed that the greater the sense of support from coworkers, the greater the self‐efficacy to continue breastfeeding and the more likely to continue breastfeeding (Zhuang et al., 2019).

Most of the nurses in our study were work‐experienced primary workforce and the nurses reported that they were still more capable of working than nurses of lower seniority, even though they had just returned to work and were still not quite familiar with their jobs. However, the policy states that nurses returning to work after maternity leave have 1 h to breastfeed during the workday and cannot be scheduled for night shifts until the child is 1 year old. All these present managers with the challenge of managing the allocation of human resources. Nurses of reproductive age occupy a large part of the nursing group, and the management of those who return to their posts after delivery is often informal (Stumbitz et al., 2018). The lack of nursing staff is still a global problem. Reasonable allocation of returning nurses to maximise their value to alleviate the shortage of nurses, for example, by using flexible work arrangements for returning nurses (Hulcombe et al., 2020). Therefore, the management of returning nurses after maternity leave can be studied in‐depth in the future.

### 5.1 Implications for nursing management

Based on the results of this study, nursing managers need to be aware of the inadaptation of nurses returning to work and the need for breastfeeding. Before breastfeeding nurses return to work, managers should understand their physical and emotional changes and develop a training program to help them get through the adjustment period as soon as possible. Managers should also provide concealed expressing breast milk rooms for nurses in the workplace. When arranging work, managers should allocate relatively flexible posts and flexible breastfeeding leave rest methods for nurses and provide adequate feeding time to reduce the breastfeeding burden on nurses. In addition, managers and nurses jointly create a working atmosphere to support breastfeeding and improve work enthusiasm. Finally, all policies aimed at improving the work environment of breastfeeding nurses should be reviewed.

### 5.2 Limitations

This study still had some limitations. Purposive sampling was used for the sampling method, expecting to achieve maximum sample differentiation. However, this study lacks nurses with a master's degree and above and a supervisor nurse and above, which may lead to incomplete findings. Because of the specific policies and cultural context in China, the results of this study may not apply to nurses returning to work after maternity leave in countries other than China.

## 6 CONCLUSION

This study highlighted the work experience of breastfeeding nurses returning to work after maternity leave in Shenyang, China. It revealed the existence of emotional, physical and work changes for breastfeeding nurses returning to work, the need for a supportive work environment and the support of coworkers, managers, organisations and ownself. Helping nurses through the adjustment period of returning to work and the proper management of staff can be further studied.

## AUTHOR CONTRIBUTIONS

WXL, PYL: Study design; WXL, YJY: Data collection; WXL, YJY: Data analyses; WXL, PYL: Manuscript preparation.

## FUNDING INFORMATION

This research received no specific grant from any funding agency in the public, commercial, or not‐for‐profit sectors.

## CONFLICT OF INTEREST STATEMENT

The authors declare that they have no conflict of interest.

## ETHICAL CONSIDERATIONS

This study was approved by The Fourth Affiliated Hospital of China Medical University Ethics Committee (Decision Number: EC‐2022‐KS‐002).

## Data Availability

The data that support the findings of this study are available from the corresponding author upon reasonable request.
